# The impact of Mediterranean diet on coronary plaque vulnerability, microvascular function, inflammation and microbiome after an acute coronary syndrome: study protocol for the MEDIMACS randomized, controlled, mechanistic clinical trial

**DOI:** 10.1186/s13063-021-05746-z

**Published:** 2021-11-12

**Authors:** Ana I. Fernández, Javier Bermejo, Raquel Yotti, Miguel Ángel Martínez-Gonzalez, Alex Mira, Uri Gophna, Roger Karlsson, Reem Al-Daccak, Irene Martín-Demiguel, Enrique Gutiérrez-Ibanes, Dominique Charron, Francisco Fernández-Avilés, on behalf of the MEDIMACS research team, Ana I. Fernández, Javier Bermejo, Raquel Yotti, Enrique Gutierrez-Ibanes, Álvaro Gabaldón-Badiola, Irene Martín-Demiguel, Ricardo Sanz, Pablo Martínez-Legazpi, Jaime Elízaga, Francisco Fernández-Avilés, Elena Jurado, Miguel Ángel Martínez-Gonzalez, Cristina Razquin, Zenaida Vázquez-Ruiz, Alex Mira, Aránzazu López, Maria D. Ferrer, Uri Gophna, Leah Reshef, Roger Karlsson, Edward Moore, Göran Karlsson, Anna Winqvist, Reem Al-Daccak, Dominique Charron

**Affiliations:** 1 Department of Cardiology, Hospital General Universitario Gregorio Marañón, Facultad de Medicina, Universidad Complutense de Madrid, Instituto de Investigación Sanitaria Gregorio Marañón, and CIBERCV, Madrid, Spain; 2 Department of Preventive Medicine and Public Health, University of Navarra, IDISNA, CIBEROBN, Pamplona, Spain; 3 Department of Nutrition, Harvard TH Chan School of Public Health, Boston, USA; 4 Department of Health and Genomics, Center for Advanced Research in Public Health, CSISP-FISABIO, and CIBERESP, Valencia, Spain; 5 Department of Molecular Microbiology and Biotechnology, George S. Wise Faculty of Life Sciences, Tel Aviv University, Ramat Aviv, Tel Aviv, Israel; 6 Department of Infectious Diseases, Institute of Biomedicine, Sahlgrenska Academy of the University of Gothenburg; Sweden Nanoxis Consulting AB; Centre for Antibiotic Resistance Research (CARe), University of Gothenburg, Gothenburg, Sweden; 7 Institut National de la Santé et de la Recherche Médicale (INSERM) UMRS-97f, Université Paris-Diderot, HLA et Médecine, Labex Transplantex, Hôpital Saint-Louis, Paris, France

**Keywords:** Atherosclerosis, Mediterranean diet, Immune system, Microbiota, -Omics, Randomized controlled trial

## Abstract

**Background:**

Primary prevention trials have demonstrated that the traditional Mediterranean diet is associated with a reduction in cardiovascular mortality and morbidity. However, this benefit has not been proven for secondary prevention after an acute coronary syndrome (ACS). We hypothesized that a high-intensity Mediterranean diet intervention after an ACS decreases the vulnerability of atherosclerotic plaques by complex interactions between anti-inflammatory effects, microbiota changes and modulation of gene expression.

**Methods:**

The MEDIMACS project is an academically funded, prospective, randomized, controlled and mechanistic clinical trial designed to address the effects of an active randomized intervention with the Mediterranean diet on atherosclerotic plaque vulnerability, coronary endothelial dysfunction and other mechanistic endpoints. One hundred patients with ACS are randomized 1:1 to a monitored high-intensity Mediterranean diet intervention or to a standard-of-care arm. Adherence to diet is assessed in both arms using food frequency questionnaires and biomarkers of compliance. The primary endpoint is the change (from baseline to 12 months) in the thickness of the fibrous cap of a non-significant atherosclerotic plaque in a non-culprit vessel, as assessed by repeated optical coherence tomography intracoronary imaging. Indices of coronary vascular physiology and changes in gastrointestinal microbiota, immunological status and protein and metabolite profiles will be evaluated as secondary endpoints.

**Discussion:**

The results of this trial will address the key effects of dietary habits on atherosclerotic risk and will provide initial data on the complex interplay of immunological, microbiome-, proteome- and metabolome-related mechanisms by which non-pharmacological factors may impact the progression of coronary atherosclerosis after an ACS.

**Trial registration:**

ClinicalTrials.govNCT03842319. Registered on 13 May 2019

**Supplementary Information:**

The online version contains supplementary material available at 10.1186/s13063-021-05746-z.

## Background

Cardiovascular morbidity and mortality are mainly associated with atherosclerosis, a systemic, chronic, dynamic, progressive and incurable condition [1]. Atherosclerosis causes coronary artery disease (CAD), the most prevalent non-communicable disease in Western countries. The first manifestation of CAD is often an acute coronary syndrome (ACS), which is a life-threatening condition. The number of hospital admissions for ACS in European countries varies between 90 and 312 per 100,000 inhabitants per year [2]. Despite the use of reperfusion therapy and intensive post-cardiac event treatments, 12% of patients admitted due to an ACS will die within the following 6 months [3].

Atherosclerosis is initiated by endothelial dysfunction [4] followed by the accumulation of cholesterol into the subendothelium of large arteries, which triggers a response cascade that results in the promotion of plaque formation. Whenever the fibrous cap that separates the plaque from the vessel lumen is broken, the highly thrombogenic plaque material is exposed to the blood, locally triggering the intracoronary thrombosis phenomena responsible for ACSs. Immune-mediated complexes are key pathophysiological elements for plaque rupture [5, 6]. Vulnerable plaques at the highest risk of rupture have a large lipid core. But most importantly, the risk of plaque rupture is inversely proportional to the thickness of this fibrous layer [7]. By facilitating plaque rupture and promoting thrombogenicity, systemic inflammation is known to increase the risk of an ACS. Moreover, beyond these local factors, coronary plaque complications frequently associate a widespread inflammation of the full coronary arterial bed [8].

After an ACS, a complex process of lesion repair and vessel healing takes place. Inflammation plays a central pathophysiological role in vessel healing [9] and is also involved in the process of ischemia-reperfusion injury and myocardial healing of the ischemic myocardium. Closely related to inflammation, gut dysbiosis is also involved in the pathogenesis of atherosclerosis by a number of mechanisms [10]. The oral cavity is the inlet of the gastrointestinal and respiratory systems and is connected extensively to the blood stream via the highly vascularized oral mucosa [11]. In addition, oral microbiota may be a factor in the early phases of atherosclerosis due to its direct involvement in vascular function [12–14].

Consequently, after an ACS, the complex interplay between inflammation, changes in microbiota and gene expression is a potential target for dietary interventions. The Mediterranean diet (MedDiet), based on the traditional dietary pattern found in olive-growing areas [15, 16], is particularly well suited for this purpose. The traditional MedDiet reduces cardiovascular mortality [17, 18]. The Lyon randomized trial [19] and the large primary-prevention PREDIMED trial have demonstrated that a MedDiet intervention reduces the incidence of major cardiovascular events [20, 21]. Several molecular mechanisms seem to be involved in the risk-reduction effect of MedDiet in primary cardiovascular prevention [22–26]. Epidemiological evidence [27] and clinical trials [28] suggest that cardiovascular protection is achieved by consuming either extra-virgin olive oil (EVOO) or nuts, together with an emphasis in plant-derived foods—key elements of the MedDiet. Moreover, it improves endothelial function and reduces blood pressure compared to a low-fat diet [29]. MedDiet also contains nitrate-rich green leafy vegetables, which have been shown to improve cardiovascular parameters [30]. Although this evidence anticipates a potential beneficial effect of a high intense MedDiet intervention after an ACS, a comprehensive mechanistic understanding of how MedDiet influences cardiovascular risk and the atherosclerotic substrate is lacking. The effect of a MedDiet intervention on the microbiota and the immune system and its potential for secondary prevention also remains unknown. This information could be of great value not only to support clinical prescription of traditional MedDiet after an ACS, but also to shed light on the molecular pathways involved in the natural history of atherosclerosis and, eventually, to identify new targets for preventing disease progression.

## Methods/design

The MEDIMACS clinical trial is designed on the global hypothesis that a comprehensive view of the biological processes triggered by diet nutrients provides new insights into the roles of the immune response and microbiome-related metabolism on coronary artery disease progression, plaque stabilization and coronary physiology. To test this hypothesis, we use an experimental randomized clinical trial design. The alternative hypothesis is that, compared to standard of care, a high-intensity MedDiet intervention after an ACS increases the fibrous cap thickness of atherosclerotic plaques. Fibrous cap thickness has been demonstrated to be a major determinant of plaque vulnerability, and it is a sensitive surrogate of cardiac ischemic events in the long term [31].

The MEDIMACS study is a 1:1 prospective, randomized, controlled, mechanistic and multidisciplinary clinical trial designed to address the effects of a 12-month intensive dietary intervention based on the traditional MedDiet on atherosclerotic plaque vulnerability and coronary endothelial dysfunction. Researchers who will address the primary outcome are blinded to the intervention assignment. Patients are being recruited in a single tertiary hospital in Spain.

### Study objectives

The general aim of the MEDIMACS trial is to determine the immunological, microbiome-related, proteomic, metabolomic mechanisms by which the Mediterranean diet impacts the progression of coronary atherosclerosis in patients after an ACS. The principal objective of the clinical trial is to address the effect of the Mediterranean diet on coronary plaque vulnerability after an ACS. Secondary objectives are (1) to determine the role of MedDiet on microbiome-related inflammation, nitric oxide metabolism, oxidative stress, choline metabolism and lipid metabolism; (2) to define molecular pathways responsible for interplay between diet, microbiota and atherosclerotic plaque progression and stabilization through a personalized evaluation of the immune, proteomic and metabolomic profiles; (3) to propose candidate biomarkers for patient stratification and prediction of the diet response; and (4) to propose microbiome-targeted therapeutic strategies in secondary prevention after an ACS.

### Participants

Patients admitted at the Hospital General Universitario Gregorio Marañón, Madrid, for an ACS fulfilling inclusion criteria (Table 1) are prospectively enrolled in the study within the first 72 h after hospital admission.

**Table 1:** Inclusion and exclusion criteria for the MEDIMACS clinical trial

Inclusion criteria	
1. Equal or older than 18 years	
2. An established diagnosis of ACS based on clinical, electrocardiographic and laboratory criteria	
3. An intermediate (20–60% lumen stenosis) atherosclerotic lesion identified in a non-culprit coronary artery with diameter > 2.0 mm (target lesion) not undergoing percutaneous coronary intervention at the time of the revascularization procedure	
4. Willingness to modify his/her diet	
5. Able to follow up and answer questionnaires	
Exclusion criteria	
1. Thrombolysis in Myocardial Infarction (TIMI) score < 3 in the culprit vessel (unsuccessful revascularization)	
2. Killip classification III/IV	
3. Active systemic infection, chronic inflammatory disease, periodontal disease or treatment with corticosteroids, antibiotics or immunomodulators within the past 3 months	
4. Renal insufficiency with glomerular filtration less than 30 mL/min	
5. Severe hepatic insufficiency (liver cirrhosis in Child B or C stages)	
6. Any comorbidity with life expectancy of less than 1 year	

**Fig. 1:**
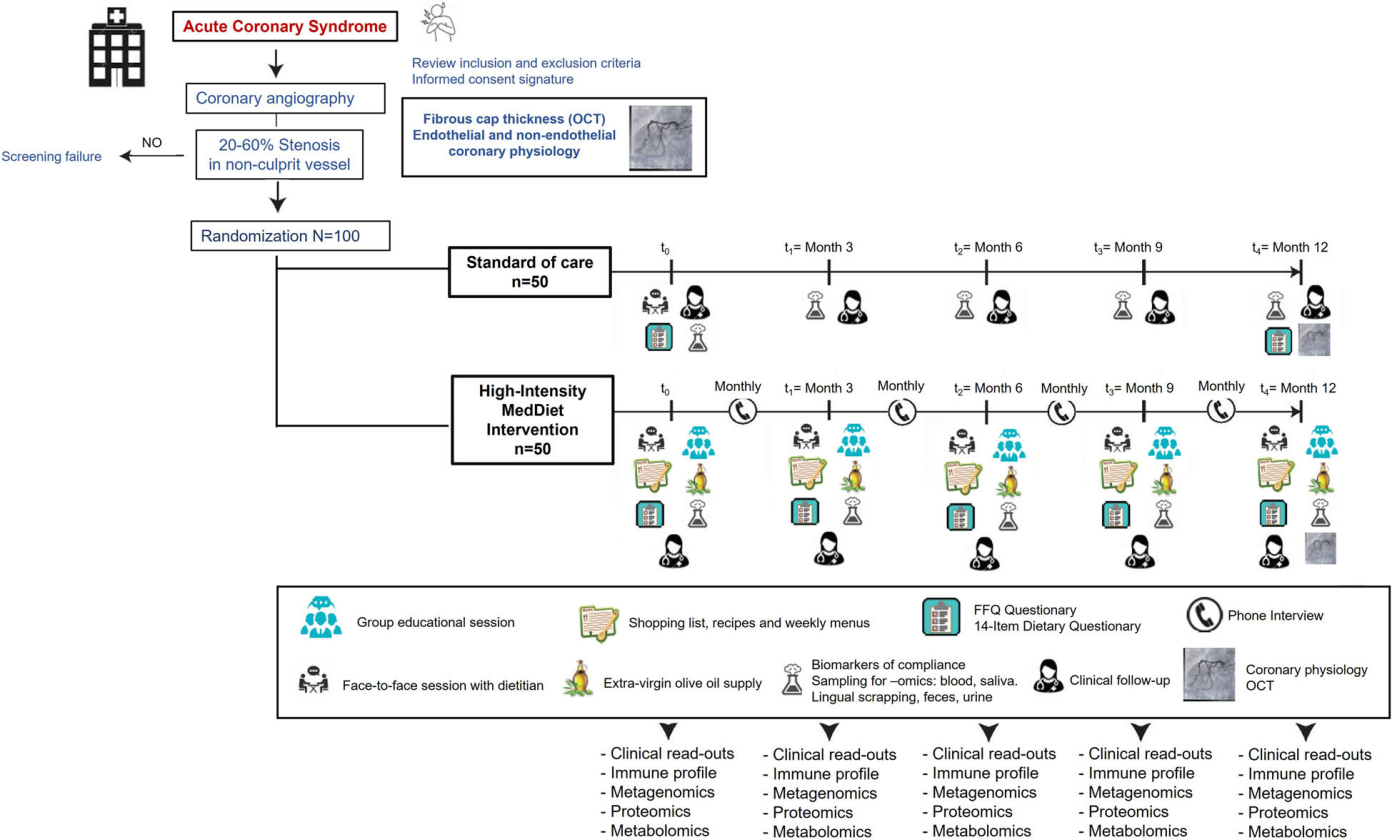
The MEDIMACS trial overview

Trial overview and allocation are shown in Fig. 1. Before undergoing coronary angiography (performed on clinical grounds), patients are informed by clinical cardiologists about the research project and invited to sign the informed consent document. Coronary intervention procedures are indicated. The MEDIMACS intracoronary research diagnostic study is initiated after completing the revascularization procedure of the culprit coronary lesion, when indicated. Only patients showing an intermediate lesion (20–60% of lumen area) in a non-culprit vessel are finally enrolled in the study (see the “Inclusion criteria” section). This intermediate lesion is labelled as the target lesion for the measurement of the primary endpoint. Within the following 12 h, patients are randomized to receive either a high-intensity intervention to promote the traditional MedDiet or standard-of-care recommendations for 12 months.

### Randomization

Patients will be 1:1 randomly allocated into two groups, intensive MedDiet intervention or standard of care. Randomization is conducted blindly by means of an Internet-based, computer-generated random-number system. The Department of Preventive Medicine and Public Health of the University of Navarra is responsible for the randomization procedure. Participants are stratified by sex and age (< 66, 66–75, > 76 years). The system automatically allocates each participant to their assigned group according to a random and non-predictive algorithm, out of control of any staff involved in the trial. To obtain approximately equally sized groups, the random generation system is adaptive, meaning that the probability of treatment assignment changes according to assigned treatments of patients already in the trial. This implies recalculation of treatment assignment probabilities for each new patient.

### Diet intervention

#### Intervention group

Patients allocated to the high-intensity MedDiet intervention group (Fig. 1) are individually interviewed by a dedicated dietitian and participate in individual and group sessions at enrolment and at 3, 6, 9 and 12 months. A personalized MedDiet with detailed nutritional composition and total energy intake is adapted to participant’s weight, age and requirements and the dietitian advice for his/her individual needs. The high-intensity MedDiet indications promote the consumption of olive oil, fresh fruits, vegetables, nuts, legumes and fish. A detailed list of indications is specified in Additional file 1. Patients in this group are provided with 4 l per month of free EVOO during the 12-month follow-up period and undergo monthly phone-call follow-up interviews. Recipes, shopping lists and weekly menus are provided to optimize adherence to the traditional MedDiet, replicating the PREDIMED clinical trial [32].

#### Control group

For ethical reasons, in patients allocated to the control group (standard of care), MedDiet is being recommended as part of the secondary prevention programme that all patients receive after an ACS. Diet recommendations are included in the hospital discharge report, focused on the advantages of the currently used Spanish MedDiet eating pattern. Briefly, the Spanish MedDiet eating pattern also emphasizes the consumption of olive oil, wheat, wine, fresh fruits, vegetables, nuts, rice, legumes and fish and seafood; limited consumption of meat and dairy products; and very limited consumption of cream, butter or margarine sugary drinks, baked goods, industrial pastries and pre-cooked foods or dishes. Although these recommendations are common to both intervention arms, previous evidence demonstrates that in the absence of intensive interventions compliance is limited [33]. Thus, significantly different dietary outcomes are anticipated between the two groups.

Implementing standard-of-care diet recommendations or the MedDiet advice does not require alteration to usual care pathways, including the use of any medication, and these will continue for both trial arms.

### Participant timeline and assessments

**Fig. 2:**
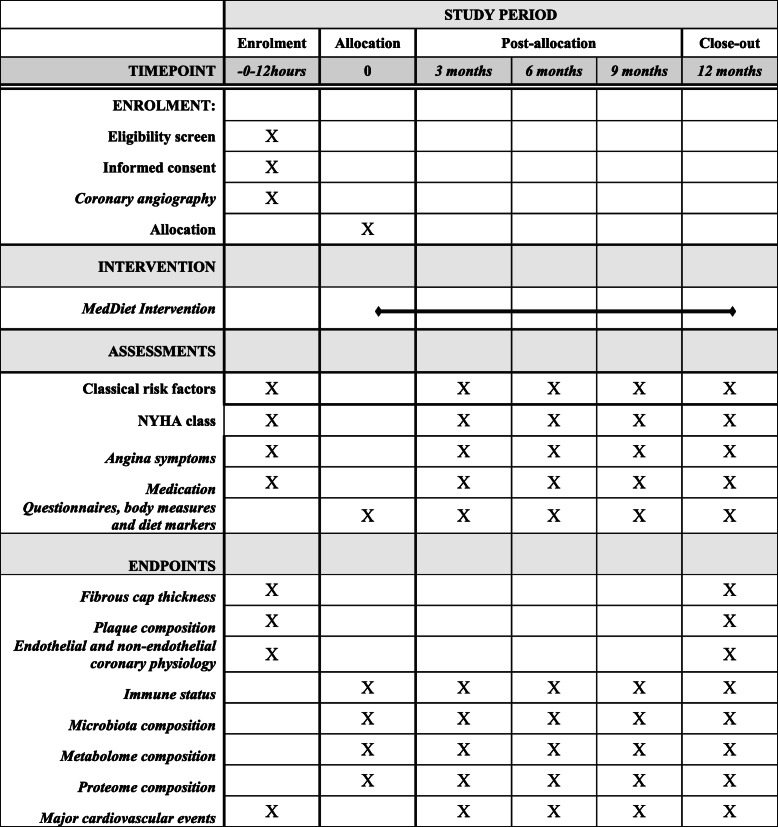
The MEDIMACS schedule of enrolment, intervention, assessments and endpoints in accordance with the SPIRIT guideline

The schedule of enrolment, interventions and assessments in accordance with SPIRIT is outlined in Fig. 2.

All patients undergo a complete clinical evaluation at baseline and at 3, 6, 9 and 12 months including a careful assessment of classical cardiovascular risk factors (blood pressure, lipid profile, glycaemic control, medication and alcohol and tobacco consumption). In each visit, the New York Health Association functional class, angina symptoms and major cardiovascular events (cardiac death, need for coronary revascularization and hospital admission for acute coronary syndrome) are recorded. In both groups, the food frequency questionnaire (FFQ) and the Mediterranean Diet Adherence Screener (MEDAS—see below) dietary screener are filled, as well as exercise, nitrate consumption and dental hygiene questionnaires are completed at baseline and at 12 months. In the intervention group, these questionnaires are also completed at the 3-, 6- and 9-month follow-up visits. At the end of the 12-month follow-up period, patients will undergo a repeated intracoronary procedure with measurements of optical coherence tomography (OCT) and endothelial function in the target lesion (see Additional file 2). All patients are sampled for blood, urine, saliva, lingual scrapping and whole faeces at enrolment and at 3, 6, 9 and 12 months. Samples are processed and stored at −80 °C for determination of immune status, protein and metabolite profiles and oral and gastrointestinal microbiota (see Additional file 2).

### Assessment of adherence to diet

A detailed plan to quantify compliance to MedDiet in both arms has been designed (Fig. 1) based on the analysis of the 137-item validated food frequency questionnaire (FFQ) and a 14-item dietary screener (MEDAS) [34]. Moreover, biological markers of compliance in a random sample of 25% of the participants will be used to blindly ascertain compliance. Plasma and urine samples will be used to determine plasma fatty acid composition, urinary tyrosol and hydroxytyrosol, urinary resveratrol and ethanol and plasma folate concentrations as markers of compliance [32].

### Outcomes

The primary endpoint of the MEDIMACS trial is the change (from baseline to month 12) in fibrous cap thickness of an intermediate atherosclerotic plaque in a non-culprit vessel (identified as the target lesion), as measured by OCT. OCT allows for a very accurate measurement of fibrous cap thickness, as demonstrated in histological validation studies [35]. Moreover, OCT allows the quantitation of plaque composition, lesion size, thickness of the fibrotic layer, lipid arch, minimal lumen area, calcification, macrophage density and plaque rupture [36]. All measurements of OCT are performed by an external core lab, blinded to the allocated diet group.

Secondary endpoints of the MEDIMACS trial are:

1. Changes (baseline to month 12) in the minimum luminal area (mm^2^) and intraluminal diameter measured by OCT in the target lesion
2. Presence of anatomic findings related to plaque instability and vulnerability at 12 months identified by OCT in the target lesion (atheroma plaque rupture, erosion or thrombus)
3. Changes (baseline to month 12) in the micro- and macrovascular endothelial function as a marker of early stages of coronary atherosclerosis, measured by a combined flow-pressure guidewire in the target lesion (change in maximum average speed, coronary flow, coronary flow reserve, fractional flow reserve, basal and hyperaemia microvascular resistances, resistance of basal stenosis and hyperaemia, and coronary impedance during the cardiac cycle)
4. Differences between groups in the micro- and macrovascular endothelial function at 12 months
5. Differences between groups in the neointima thickness of the stent in the treated artery (at 12 months)
6. Changes (baseline to month 12) of the immunological status, including innate T and B cell populations (NK, innate lymphoid cells, monocytes/macrophages) and NKT cells
7. Changes (baseline to month 12) of gene expression of cytokines and epigenetic profiles associated with methylation, histidine decarboxylase and miRNA
8. Changes (baseline to month 12) in the diversity of the T cell receptors using massive sequencing methodologies
9. Changes (baseline to month 12) of the microbiota abundance and diversity identified using the 16S rRNA massive sequencing approach and the functional analysis of complete metagenomes
10. Changes (baseline to month 12) of the host and microbiota protein and metabolomics profiles using mass spectrometry (MS) and nuclear magnetic resonance (NMR)-based methods
11. Incidence at 1 year of major adverse cardiovascular events (MACE) defined as cardiovascular death, myocardial infarction or ischemic stroke
12. Clinically driven coronary revascularization
13. Kaplan-Meier analysis of MACE (as defined above) and of clinically driven coronary revascularization

### Adverse outcomes

All research staff will report any health problems related to the study at any time. All serious adverse events, whether associated or not with the intervention, will be documented on paper and electronic case report forms. All adverse events will be noted in the publication of the results. An analysis of clinical outcomes after completion of the 12-month follow-up period of the first 50 recruited patients is planned and approved by the Ethical Committee of the Hospital Gregorio Marañón. Although unlikely, they may recommend stopping the clinical trial if relevant safety differences are detected between groups.

### Sample size

The sample size of the MEDIMACS clinical trial has been calculated on the assumption of an expected difference of 40% on the relative change in the fibrous cap thickness of the atherosclerotic plaque from baseline to 12 months between patients undergoing a high-intensity MedDiet intervention in comparison with the standard of care. This magnitude is a 10% lower than the difference previously reported using a similar design in a clinical trial comparing the effect on the fibrous cap of lipid-lowering therapy with 5 mg/day versus 20 mg/day atorvastatin, with an estimated standard deviation of 58% [37]. Based on a two-sample bilateral alpha error of 0.05 and a power of 0.80, the required number of patients is 35 per group. Assuming an attrition rate of 10%, 100 patients are needed to be randomized following a 1:1 allocation ratio. With this sample size, different scenarios could be contemplated under potential deviations from the expected effect size (Table 2).

**Table 2:** Alternative scenarios for statistical power with *n* = 100 sample size, assuming 10% attrition rates

Effect size	Power	Comment
50%	100%	Effect addressed in EASY-FIT Study
**40%**	**99.60%**	**Baseline assumptions**
30%	93.45%	Effect 25% relatively lower than expected

Regarding the suitability of this sample size to evaluate the secondary endpoints, micro- and macrovascular endothelial functions are proven to be highly sensitive to detect meaningful effects using small sample populations (50–100 patients) [38, 39]. Microbiota, immunological and metabolic changes in response to diet interventions have been widely reported using small sample populations (20–50 patients), particularly under pathological conditions [40, 41].

### Statistical analysis plan

The primary endpoint will be analysed by intention to treat using a linear mixed-effects model accounting for repeated measures. Values of cap thickness will be entered as the dependent variable. The visit, the treatment group and their interaction will be entered as fixed effects, whereas the patient identification is entered as random effect. Thus, the coefficient of the term accounting for the interaction between the treatment group and visit provides an adjusted estimate of the magnitude of change in thickness at 1 year attributable to the effect of the intervention. The 95% confidence interval of this estimate will be assessed by asymptotic and bootstrap methods. Under a missing at random assumption, this method gives unbiased estimates of the effect. Quantitative indices of endothelial function, coronary physiology and additional metrics of plaque composition will be also analysed using linear mixed-effects models. The analysis of clinical outcomes will be conducted using the Kaplan-Meier method and the log-rank test to compare both intervention groups. Statistical comparisons will be 2-tailed and a statistical significance level of 0.05 shall be considered. Uni- and multivariate linear and non-linear mixed and regression models, adjusting for covariables, will be conducted for the relationship of microbiota composition (diversity and abundance metrics), the number and type of immune cell populations, proteins and metabolites and diet and clinical outcomes.

A modified intention-to-treat analysis including all randomized patients who undergo the catheterization procedure at a 12-month follow-up visit will be used to evaluate the primary endpoint and all secondary endpoints (*intention-to-treat analysis set*). In addition, a per-protocol analysis will be performed following recent directions for analyses of pragmatic trials to control for confounding [42] after excluding from the intervention arm those patients in whom the food frequency questionnaires and biological markers indicate low compliance to the prescribed MedDiet (*per-protocol set*). Secondary endpoints #4–10 will be analysed in all patients that complete the 12-month follow-up period regardless if they undergo the follow-up catheterization procedure or not (*full analysis set*).

## Discussion

Diet is one of the main environmental factors triggering atherosclerosis and plaque progression. In fact, the current “American Dietary Guidelines’ Key Recommendations for Healthy Eating” recommends a Mediterranean-style pattern as one of its three suggested healthy diet styles [43]. Moreover, when changes over time are assessed, improved adherence to the traditional Mediterranean-style pattern is strongly associated with reductions in all-cause mortality and cardiovascular mortality [44]. On this basis, we have designed the MEDIMACS clinical trial to understand the mechanisms involved in diet-induced changes on anatomical and functional biomarkers of coronary atherosclerosis.

Several individual nutrients have been mechanistically associated with beneficial effects on the gastrointestinal tract microbiome. However, in contrast to the isolated single-nutrient approach, the MedDiet intervention is based on current paradigms of studying overall dietary patterns. These have the advantage of taking into consideration the potential synergistic or antagonistic effects of different nutrients and better represent the dietary practices found in real-world diets [45]. The appropriateness of this intervention is also supported by well-established cardiovascular benefits in primary prevention. Although several large-scale trials using MedDiet interventions are currently ongoing, to the best of our knowledge, no trial is currently including patients in the subacute phase after an ACS.

Numerous molecular pathways have been involved in the beneficial effect of MedDiets, such as reduction in the expression of endothelial leukocyte adhesion molecules and reduction of DNA synthesis in human coronary smooth muscle cell. However, the link between MedDiet and cardiovascular protection has not been fully elucidated. Remarkably, the interplay between diet, gut and oral microbiota and the temporal changes of plaque composition and vascular function—key agents in the pathogenesis of atherosclerosis—remains largely unknown.

### Clinical impact

The combination of a well-controlled experimental intervention and complex mechanistic objectives shall provide valuable insights into protective microbiota-mediated roles in the MedDiet. Moreover, the identification of specific microbial profiles related to cardiovascular health could help to design probiotic treatments―alone or in combination with food supplements―to improve their efficacy. In addition, microbiota-based diagnostic kits to personalize and monitor cardiovascular risk could be developed. In the future, tests of microbiota-mediated markers could identify individuals at a particular cardiovascular risk who should be prioritized for prevention.

Shifting dietary patterns and directions worldwide entails substantial economic and public health efforts that are frequently in overt conflict with the major stakeholders of food companies. Further demonstrating the efficacy of MedDiet for secondary prevention in patients with CAD may help to justify the need and opportunity of clinical interventions to promote adherence to this traditional diet. Furthermore, a comprehensive view of the biological processes triggered by diet nutrients could eventually lead to the design of diet modifications based on well-established biological pathways.

### Limitations

Complying with basic ethical principles, the control group will receive standard-of-care diet recommendations which include several aspects of the MedDiet. Other alternatives, such as a low-fat diet, are associated with worse outcomes than MedDiet and would not be ethical to implement in a contemporary clinical trial [3]. We recognize that, for the purpose of MEDIMACS, adherence to MedDiet in the control group may blur the effect of the experimental intervention and decrease the power to detect differences between arms. However, it has been demonstrated that a personalized, professionalised and closely monitored high-intensity intervention is highly effective to promote adherence to traditional MedDiet, whereas general recommendations are much less powerful [20]. Therefore, we anticipate that true adherence to MedDiet specifications will be highly different between both groups. Nevertheless, primary and secondary endpoints will be analysed both by intention-to-treat and by a per-protocol basis. For this purpose, ad hoc biomarkers have been implemented and will provide investigators with robust metrics of diet adherence and efficacy.

The primary endpoint of the trial is based on intracoronary imaging, and therefore, an invasive catheterization procedure is required for obtaining this read-out. Because patients may refuse undergoing this second study, a 45% attrition has been pre-specified. A detailed plan to quantify compliance to MedDiet in both arms has been designed, including validated dietary self-reporting questionnaires and biomarkers. Biomarkers of compliance will be measured in random subset of participants from the two arms of the trial as a potential way of accommodating both systematic and random components of measurement error in dietary self-reporting.

## Trial status

Protocol version 4.0. Date: November 10, 2018

Date recruitment started: May 14, 2019

Recruitment completed: August 2, 2021

Recruitment status: Completed

## Supplementary Information

**Additional file 1.** High-intensity MedDiet specifications.

**Additional file 2.** Methods for the determination of the study variables.

## Data Availability

Data generation and analysis procedures follow the FAIR data management principles of Findability, Accessibility, Interoperability and Reusability. An eCRF has been built, using a collaborative platform (RedCap) administered by the principal investigator, in which all demographic, analytical and clinical data will be stored. A sequential code number will be automatically assigned to each participant. Personal data will not be included in the research database. Confidentiality of patient data will be maintained throughout the study; the list of codes for each participant will be stored securely and will be accessible only to the principal investigator. Trial data will be accessible only to the research team. An in-house certified research nurse not participating in the study is dedicated to external monitoring following Standard Operating Procedures. Sequences, genotypes and protein and immune profiles will be organized and indexed in metadata files following a unique code of assignment that will be submitted for releasing to public database repositories such as GenBank, Gene Expression Omnibus and ClinicalTrials.gov, following the International Standards for Genomes and Metagenomes. MEDIMACS researchers embrace the values of openness and transparency in science and will publish the results of this study within 6 months after trial completion. The datasets generated and/or analysed during the current study will be made available upon request to the investigators and after approval by all the members of the MEDIMACS consortium.

## Abbreviations

ACS: Acute coronary syndrome
TMAO: *N*-oxide trimethylamine
MedDiet: Mediterranean diet
EVOO: Extra-virgin olive oil
FFQ: Food frequency questionnaire
MEDAS: Mediterranean Diet Adherence Screener
OCT: Optical coherence tomography
MACE: Major adverse cardiovascular events
