# Supplementary material for: The impact of Mediterranean diet on coronary plaque vulnerability, microvascular function, inflammation and microbiome after an acute coronary syndrome: study protocol for the MEDIMACS randomized, controlled, mechanistic clinical trial
Source: Trials. 2021 Nov 12;22:795. doi: 10.1186/s13063-021-05746-z (PMC8588729; doi:10.1186/s13063-021-05746-z)
Supplement: Supplementary file 1 — Additional file 1. High-intensity MedDiet specifications. [file 13063_2021_5746_MOESM1_ESM.docx]

**High-intensity MedDiet specifications:**

High-intensity MedDiet intervention includes the following indications:

1. Abundant (> 40 g/d) use of extra-virgin olive oil (EVOO) as the only culinary fat for cooking, and for using as a spread and for dressing dishes.
2. Consumption of > 2 daily servings of vegetables, at least one of them as a salad
3. Consumption of ≥ 3 daily servings of fresh fruit
4. Consumption of ≥ 3 weekly servings of legumes
5. Consumption of ≥ 3 weekly servings of fish or seafood
6. Consumption of ≥ 3 weekly servings of mixed tree nuts
7. Preferred white (poultry) instead of processed or red meats (lamb, veal, pork)
8. Cook regularly with tomato, garlic and onion adding or no other aromatic herbs, and dress vegetables, pasta, rice and other dishes with tomato, garlic and onion (with or without additional aromatic herbs).
9. Cream, butter, margarine, cold meat, paté, duck, carbonated and/or sugared beverages, pastries, industrial bakery products, cakes and biscuits industrial desserts, French fries or potato chips, and out-of-home pre-cooked cakes and sweets are not allowed.
10. Two main meals per day should be eaten (seated at a table, lasting more than 20 minutes).
11. For usual drinkers, the main source of alcohol should be wine (maximum 300 mL per day) consumed only with meals on a daily basis; if wine intake is unusual, patients are recommended to take a single glass of wine per day (150 mL for men, 100 mL for women) during the main meal.
12. Ad libitum consumption is allowed for the following food items: nuts, eggs, fish, seafood, legumes, fruit, vegetables, low fat cheese and whole-grain cereals.
13. Limited consumption (< 1 serving per week) is advised for cured ham, red meat (after removing all the visible fat), chocolate (only dark chocolate, with more than 50% cocoa), as well as cured or fatty cheeses.
