# Supplementary material for: The impact of Mediterranean diet on coronary plaque vulnerability, microvascular function, inflammation and microbiome after an acute coronary syndrome: study protocol for the MEDIMACS randomized, controlled, mechanistic clinical trial
Source: Trials. 2021 Nov 12;22:795. doi: 10.1186/s13063-021-05746-z (PMC8588729; doi:10.1186/s13063-021-05746-z)
Supplement: Supplementary file 2 — Additional file 2. Methods for the determination of the study variables. [file 13063_2021_5746_MOESM2_ESM.docx]

## **Study variables**

### *Fibrous cap thickness of atherosclerotic plaques*

Fibrous cap thickness and quantitation of plaque composition, lesion size, thickness of the fibrotic layer, lipid arch, minimal lumen area, calcification, macrophage density, and plaque rupture will be measured by OCT.

### *Endothelial and non-endothelial coronary physiology*

Macrovascular and microvascular endothelial function of the non-culprit artery will invasively assessed, as well as the non-endothelial microvascular function at baseline and 12-months.

Endothelial responses will be provoked by slow intracoronary infusions of acetylcholine (1) and the non-endothelial hyperemia by an intravenous perfusion of adenosine, at a dose of 140 ug/kg/min (2). The microvascular function will be measured by means of a pressure-thermodilution wire (PressureWire, Abbott), as previously described (3, 4). The macrovascular compartment responses will be assessed by coronary angiography after each acetylcholine infusion (1, 5).

### *Immune Status*

Immune cell populations will be assessed dynamically using high performance cytometry and functional immuno-analyses. Next Generation Sequencing will be used for immunogenic characterization of specific immune-related genes. Immune repertoire diversity of lymphocytes will be assessed using a home-made assay (6, 7).

### *Microbiota Composition*

Intestinal and oral microbiota will be determined using the 16S rRNA target amplicon approach for taxonomic analyses. Taxonomic and metabolic pathway analyses will be complemented by shotgun metagenome sequencing to determine the functional profiles and identify specific microorganisms related to diet and atherosclerosis (8).

### *Metabolome and Proteome Composition*

Patient protein profiles from collected plasma will be analysed using the Luminex methodology (9). Novel methods based on mass spectrometry (MS) will also be explored (10). MS-based metaproteomic analyses of faeces will be employed for proteotyping as well as for evaluation of functional pathway analyses (11). Metabolites from collected serum, urine and faeces will be analysed using both NMR- and MS-based methodologies.

Immune profiling, metagenomic, metabolomic and proteomic analyses will be conducted to generate global profiles representative of diet and immune and metabolism status. The combination of data obtained from the clinical outputs and multiomic analyses will be performed using multiple bioinformatic tools based on enrichment and overrepresentation, on network-based analyses, on pathway reconstructions and on empirical correlation analyses (12-15). Novel statistical frameworks for cross-trait penalized regression (16) will be used to identify shared causal pathways between several outcomes.

# **REFERENCES**

1. Luscher TF, Pieper M, Tendera M, Vrolix M, Rutsch W, van den Branden F, et al. A randomized placebo-controlled study on the effect of nifedipine on coronary endothelial function and plaque formation in patients with coronary artery disease: the ENCORE II study. Eur Heart J. 2009;30(13):1590-7.

2. Kern MJ, Lerman A, Bech JW, De Bruyne B, Eeckhout E, Fearon WF, et al. Physiological assessment of coronary artery disease in the cardiac catheterization laboratory: a scientific statement from the American Heart Association Committee on Diagnostic and Interventional Cardiac Catheterization, Council on Clinical Cardiology. Circulation. 2006;114(12):1321-41.

3. Pijls NH, De Bruyne B, Smith L, Aarnoudse W, Barbato E, Bartunek J, et al. Coronary thermodilution to assess flow reserve: validation in humans. Circulation. 2002;105(21):2482-6.

4. Fearon WF, Balsam LB, Farouque HM, Caffarelli AD, Robbins RC, Fitzgerald PJ, et al. Novel index for invasively assessing the coronary microcirculation. Circulation. 2003;107(25):3129-32.

5. Diez-Delhoyo F, Gutierrez-Ibanes E, Sanz-Ruiz R, Vazquez-Alvarez ME, Gonzalez Saldivar H, Rivera Juarez A, et al. Prevalence of Microvascular and Endothelial Dysfunction in the Nonculprit Territory in Patients With Acute Myocardial Infarction. Circ Cardiovasc Interv. 2019;12(2):e007257.

6. Talvensaari K, Clave E, Douay C, Rabian C, Garderet L, Busson M, et al. A broad T-cell repertoire diversity and an efficient thymic function indicate a favorable long-term immune reconstitution after cord blood stem cell transplantation. Blood. 2002;99(4):1458-64.

7. Farge D, Henegar C, Carmagnat M, Daneshpouy M, Marjanovic Z, Rabian C, et al. Analysis of immune reconstitution after autologous bone marrow transplantation in systemic sclerosis. Arthritis Rheum. 2005;52(5):1555-63.

8. Belda-Ferre P, Alcaraz LD, Cabrera-Rubio R, Romero H, Simon-Soro A, Pignatelli M, et al. The oral metagenome in health and disease. ISME J. 2012;6(1):46-56.

9. Surenaud M, Manier C, Richert L, Thiebaut R, Levy Y, Hue S, et al. Optimization and evaluation of Luminex performance with supernatants of antigen-stimulated peripheral blood mononuclear cells. BMC Immunol. 2016;17(1):44.

10. Yates JR, 3rd. Recent technical advances in proteomics. F1000Res. 2019;8.

11. Kolmeder CA, Salojarvi J, Ritari J, de Been M, Raes J, Falony G, et al. Faecal Metaproteomic Analysis Reveals a Personalized and Stable Functional Microbiome and Limited Effects of a Probiotic Intervention in Adults. PLoS One. 2016;11(4):e0153294.

12. Boulund F, Karlsson R, Gonzales-Siles L, Johnning A, Karami N, Al-Bayati O, et al. Typing and Characterization of Bacteria Using Bottom-up Tandem Mass Spectrometry Proteomics. Mol Cell Proteomics. 2017;16(6):1052-63.

13. McHardy IH, Goudarzi M, Tong M, Ruegger PM, Schwager E, Weger JR, et al. Integrative analysis of the microbiome and metabolome of the human intestinal mucosal surface reveals exquisite inter-relationships. Microbiome. 2013;1(1):17.

14. Kurtz ZD, Muller CL, Miraldi ER, Littman DR, Blaser MJ, Bonneau RA. Sparse and compositionally robust inference of microbial ecological networks. PLoS Comput Biol. 2015;11(5):e1004226.

15. Ma W, Huang C, Zhou Y, Li J, Cui Q. MicroPattern: a web-based tool for microbe set enrichment analysis and disease similarity calculation based on a list of microbes. Sci Rep. 2017;7:40200.

16. Chung W, Chen J, Turman C, Lindstrom S, Zhu Z, Loh PR, et al. Efficient cross-trait penalized regression increases prediction accuracy in large cohorts using secondary phenotypes. Nat Commun. 2019;10(1):569.
